# Robust Conditional Independence maps of single-voxel Magnetic Resonance Spectra to elucidate associations between brain tumours and metabolites

**DOI:** 10.1371/journal.pone.0235057

**Published:** 2020-07-01

**Authors:** Raúl Vicente Casaña-Eslava, Sandra Ortega-Martorell, Paulo J. Lisboa, Ana Paula Candiota, Margarida Julià-Sapé, José David Martín-Guerrero, Ian H. Jarman

**Affiliations:** 1 Department of Applied Mathematics, Liverpool John Moores University (LJMU), Liverpool, United Kingdom; 2 Centro de Investigación Biomédica en Red en Bioingeniería, Biomateriales y Nanomedicina (CIBER-BBN), Cerdanyola del Vallès, Spain; 3 Departament d’Enginyeria Electrònica - ETSE, Universitat de València (UV), Valencia, Spain; Arizona State University & Santa Fe Institute, UNITED STATES

## Abstract

The aim of the paper is two-fold. First, we show that structure finding with the PC algorithm can be inherently unstable and requires further operational constraints in order to consistently obtain models that are faithful to the data. We propose a methodology to stabilise the structure finding process, minimising both false positive and false negative error rates. This is demonstrated with synthetic data. Second, to apply the proposed structure finding methodology to a data set comprising single-voxel Magnetic Resonance Spectra of normal brain and three classes of brain tumours, to elucidate the associations between brain tumour types and a range of observed metabolites that are known to be relevant for their characterisation. The data set is bootstrapped in order to maximise the robustness of feature selection for nominated target variables. Specifically, Conditional Independence maps (CI-maps) built from the data and their derived Bayesian networks have been used. A Directed Acyclic Graph (DAG) is built from CI-maps, being a major challenge the minimization of errors in the graph structure. This work presents empirical evidence on how to reduce false positive errors via the False Discovery Rate, and how to identify appropriate parameter settings to improve the False Negative Reduction. In addition, several node ordering policies are investigated that transform the graph into a DAG. The obtained results show that ordering nodes by strength of mutual information can recover a representative DAG in a reasonable time, although a more accurate graph can be recovered using a random order of samples at the expense of increasing the computation time.

## 2 Introduction

Structure finding methods lie within the field of Probabilistic Graphical Models. They have been studied extensively [1, 2], especially from a theoretical perspective, as they offer an efficient graphical approach to apply statistical estimates in a complex system. They serve as a framework for Bayesian and Markov networks [3], and have two components: a structure in the form of a graph, and a set of parameters that can be used to make statistical estimations.

One of the goals of this work is to develop a robust method to find the structure of Bayesian Networks (BNs) that fit real-word data where the correlation structure is not known a priori, in our case comprising single voxel MR spectra from brain tumours. A BN is a probabilistic model that identifies a factorisation of the joint distribution of the data, represented in the form of a directed acyclic graph (DAG) comprising a set of variables (nodes) and their conditional dependences (edges).

The conditional dependences are expressed by conditional probabilities of child node event sets e.g. A,B as a function of a parent node event sets e.g. C, hence *P*(*A*, *B*|*C*). Two events are conditionally independent if their joint probabilities can be factorized given the conditioning set:

$$\begin{array}{c} (A⊥\;\;\;⊥B)|C\;\Leftrightarrow \;P\left(A,B|C\right)=P\left(A|C\right)\cdot P\left(B|C\right) \end{array}$$ (1)

Conditional independence is established by statistical tests that are extensions of the *χ*^2^ test, centred on the entropy of the empirical data distributions, applied with appropriate conditioning constraints. Those methods that seek independence structures are called constraint-based methods.

Mutual associations are represented by undirected edges and conditional associations by directed edges. On the other hand, directed edges are used to explicitly indicate a direction in the dependence in the conditional probabilities. For instance, if the joint probability of three variables can be factorized as:

$$\begin{array}{c} P(A,B,C)=P(C|A,B)\cdot P(B,A)=P(C|A,B)\cdot P(B|A)\cdot P(A) \end{array}$$ (2)

Then, these variables can be represented in the following graph: *A* → *B* → *C*.

The application of multiple tests of conditional independence generates a factorisation of the full joint distribution of all of the variables, which can be represented as a graph. The factorisation formally identifies the terms in Eq 3.

$$\begin{array}{c} P\left(X_{1},X_{2},\ldots ,X_{N}\right)=\prod_{i}^{N}P(X_{i}|\;parents\left(X_{i}\right)) \end{array}$$ (3)

Clearly, multiple testing increases the risk of false positives and false negatives. Moreover, the factorisation procedure is iterative, so that the order of pruning also comes into play.

The initial part of the methodology of this work will be focused on the identification of associations between variables in a dataset. The measure of association between two variables is computed through conditional independence tests, and can then be extended to all variables creating a map of conditional independence tests, henceforth Conditional Independence Maps (CI-Maps), forming a graph of undirected edges. Then, once the CI-Maps are built, the second part of the methodology will be focused on how the CI-Map edges can be oriented in a consistent way to build a BN.

The main mathematical part of this work is based on constraint-based structure finding methods developed in [4], where a variant of the PC-algorithm [5] is used. Many constraint-based structure learning algorithms, including this work, are based on the PC-algorithm, which was developed at Carnegie Mellon University, and named after its authors Peter Spirtes and Clark Glymour.

In [4], the PC-algorithm using the mutual information (MI) for testing the conditional independence was considered, adding policies to control false positives (FP) and false negatives (FN). With the MI approximation the PC-algorithm runtime is reduced considerably, being comparable with other algorithms that just use pairwise independence test, like ARACNE [6]. On the other hand, the CI-Maps obtained have a quality comparable to that obtained by hybrid methods (constraint-based combined with search-and-score for refinement), which have longer runtime, such as the MMHC model [7].

One of the novelties introduced in [4] is the ordering of the sequence in which the independence test is performed. Recalling that the test order matters, in such a way that a pruned edge (because of a positive independent test) can affect the conditioned variables of successive tests, and therefore affecting the posterior test outcomes. An ordering based on the edge MI is proposed in [4], testing the weakest edges first (TWF). The paper shows this policy improves the quality of the graphs in terms of skeleton errors and adds consistency to the PC-algorithm, in such a way it is independent of the node order. The idea of testing TWF has been reinforced in a recent paper [8], where the PC-algorithm is also used with MI as independence test.

One of the objectives of the present work is to continue the research of [4], first evaluating empirically the skeleton errors of the CI-Maps associated with the policies, and second, developing a consistent method to transform CI-maps into BN, orienting the edges independently of the node order.

The use of MI as an estimator of independence tests is due to its relation with the G-test, which can be defined as:

$$\begin{array}{c} G_{i,j|A}=2\sum_{i,j}O_{i,j|A}\text{ln}(\frac{O_{i,j|A}}{E_{i,j|A}}) \end{array}$$ (4)

where (*i*, *j*) represents a cell of a contingency table, *O*_*i*,*j*|*A*_ ≥ 0 is the observed count in a cell (*i*, *j*) under the condition *A* (evidence), *E*_*i*,*j*|*A*_ > 0 is the expected count under the null hypothesis, *ln* is the natural logarithm, and the sum of the observed count must be equal to the total expected count, *N*, which is the number of observations.

The G-test asymptotically tends to the *χ*^2^ distribution, when it is used as independence test with variable independence as null hypothesis. It can be approximated with the second order Taylor expansion of the natural logarithm around 1.

$$\begin{array}{c} \chi^{2}=\sum_{i,j}(\frac{{\left(O_{i,j|A}-E_{i,j|A}\right)}^{2}}{E_{i,j|A}}) \end{array}$$ (5)

The MI can be expressed with discrete conditional probabilities as follows:

$$\begin{array}{c} I_{i,j|A}=\sum_{i,j}P_{i,j,A}{\text{ln}}_{2}(\frac{P_{i,j|A}}{P_{i|A}P_{j|A}}) \end{array}$$ (6)

Expressing the contingency tables with discrete probabilities, *P*_*i*,*j*_ = *O*_*i*,*j*_/*N*, the G-test can be connected asymptotically with the MI and the *χ*^2^ distribution:

$$\begin{array}{c} G_{i,j|A}∼2N\text{ln}\left(2\right)\;{\tilde{I}}_{i,j|A}∼{\tilde{\chi }}_{df}^{2} \end{array}$$ (7)

where *df* represents the degrees of freedom, *df* = (|*i*| − 1) ⋅ (|*j*| − 1) ⋅ |*A*|, in terms of the number of categories for each categorical variable.

The PC-algorithm starts with a fully connected graph. Then, edges are removed between nodes (variables) based on pairwise independence tests, increasing the number of conditioned variables as the algorithm progresses. The algorithm stops when it finally converges to a stable structure forming a CI-map. During this process the following policies are applied:

- False Discovery Rate (FDR) [9], which controls False Positives, decreasing the significance level in conditional independence tests when they are applied multiple times on the same nodes. This translates to a reduced number of edges.
- False Negative Reduction (FNR) [10], which aims to avoid independence tests if they are not powerful enough. This criterion is based on a threshold of Degrees of Freedom (DoF) that depends on the desired power for the test, the sample size, and the effect size.
- The Weakest First (TWF), which affects the order in which the graph is being pruned. The outcome of the PC-algorithm is influenced by the order in which the conditional independence tests are executed. TWF sorts the nodes by mutual information (edge strength), testing first the weakest nodes tjus reducing the problem of incorrect pruning or incorrect edge dependence discovery.

Once the structure is found, the next step is to build a Directed Acyclic Graph (DAG) following the rules defined in [11]. These rules do not necessarily lead to a unique DAG, where the most general solution is a Partial Directed Acyclic Graph (PDAG).

It is well known that the PC-algorithm is sensitive to the order in which the nodes are tested [12, 13]. This problem is addressed by using a similar solution to TWF, but ordering the nodes by descending mutual information. This policy is called The Strongest First (TSF). The DAG obtained with TSF node order is compared with DAGs generated by random node order. The Bayesian networks (BNs) derived from the DAGs are assessed by the log-likelihood function with the Bayesian Information Criteria (BIC) (Eq 8), which penalizes the graph complexity.

Other studies take different approaches, such as the Complete Partially Directed Acyclic Graphs used in [14], latent variables with the Fast Causal Inference algorithm in [5], Maximal Ancestral Graphs in [15], IDA algorithm (Intervention calculus when the DAG is Absent) in [16, 17], and Essential Graph Search in [18] based on scoring random order DAGs.

The goal of this work is to present empirical evidence for best practice in setting the optimization of three parameters: the policies of False Discovery Rate (FDR) and False Negative Reduction (FNR), and the effect of node ordering when the edges are being oriented. The parameter to set-up is then used to find an optimal structure based on data bootstrapping to create multiple CI-maps with the aim to get the most prevalent structure. This procedure is then applied to magnetic resonance spectroscopy (MRS) data from human brain tumours to investigate the metabolite dependencies.

The novel contributions of this methodological approach are the following:

- Analysis of structure finding based on PC-algorithm and its dependence on design parameters, e.g. data ordering.
- Empirical parameter optimization of CI-maps removing dependence on design parameters.
- Optimisation of FDR and FNR policies to avoid proliferation of False Positives.

From the methodological point of view, this paper is divided into two main parts, the first one dedicated to assess the parameters and policies in the PC-algorithm to build feasible CI-maps and BNs, using benchmark data where the true structure is known. The second part exploits these CI-maps on brain tumour data for the extraction of a hierarchical order of variable associations (i.e. metabolites) with respect to a target variable (tumour type).

The rest of the paper is outlined as follows. After this introduction, the paper continues with a review of the related research in section 3, also giving a description of the main parameters and policies used in the PC-algorithm. Section 4 introduces the benchmark data where the true structure is known, hence used for evaluating the parameters, and also describes the brain tumour data where the methodology is applied. Section 5 presents the methodology for the assessment of parameters with benchmark data, as well as for the selection of the most representative CI-map. The achieved results are shown in section 6 and discussed in section 7. The paper ends up in section 8 with conclusions that can be drawn from the study.

## 3 Related research

This section describes three important parameters which must be taken into account in the structure learning when using the PC-algorithm, namely, FDR, FNR and the effect of node ordering. Then, assuming there is a target node (variable) as a reference point, a bootstrapping method is presented to select the most representative CI-map with reference to the target variable, so that the selected CI-map (among all bootstrapped CI-maps) preserves the most prevalent edges connected to the target-node.

### 3.1 False discovery rate

When multiple conditional independence tests are carried out, the control of FDR is necessary. Three different variants of the FDR control policy are considered [4], namely, basic, interleaved and mini-FDR:

- The default option is the basic FDR and is applied after the convergence of the PC-algorithm.
- The interleaved variant is applied after each step of the PC-algorithm, where a step implies one more node in the conditional independence tests.
- Mini-FDR is solely responsible for pruning edges in the structure.

### 3.2 False Negative Reduction

A False Negative (FN) implies a missing edge (independence) between two nodes (variables) when there is a true dependency present between the pair of nodes. The FNR policy, based on [10], avoids testing the hypothesis if the minimum power of the test is not at least 1 − *β*, with *β* being the FN ratio. This condition sets a threshold for the maximum degrees of freedom; it depends on the sample size, *β*, the test level *α*, and a desired effect size *w*. In practice, *β* and *α* are usually pre-established standard values, and the effect size *w* has to be adjusted. This is the challenging part of the FNR, since the optimal value of *w* is dependent on the data and its sample size, and therefore it is difficult to find a good value for unknown data.

In the process to find the skeleton starting with a fully connected graph, FNR avoids some tests between nodes, and therefore the edges are kept, reducing the chance of a FN. But, at the same time, FNR may produce an undesired effect if too many edges are preserved because they are not tested, either due to a wrong choice of the parameters, or because some of these edges belong to true independent nodes.

### 3.3 The effect of node ordering

The solutions (CI-maps) of the PC-algorithm are sensitive to node ordering. This work is based on the PC-implementation presented in [4], where the authors addressed this problem by ordering the nodes by mutual information, and then testing the independence on the weakest nodes first. This node order is based on the averaged mutual information between the node and the rest of nodes by pairs. This way, the PC-algorithm starts to prune the fully connected skeleton by the weakest nodes. Applying this policy, the PC-algorithm reconstructs the structure independently of the node order.

The orientation of the edges of the skeleton to produce a DAG is usually carried out by the set of rules developed in [11, 19]. These rules are also sensitive to the node order, i.e. the order in which the edges are oriented affects the final DAG. By systematically following these rules, graph acyclicity is not guaranteed, and hence, after orienting an edge the acyclity must be checked, and if the acyclity is not preserved the last edge orientation is reversed. One of the complexities orienting an acyclic graph is that it has a tree structure, hence the edge orientation of the ancestors determine the edge structure of their descendants, and a mistake made in an ancestor edge is more punitive than a mistake in a descendant edge. The number of possible edge orientations grows non-linearly with the degree of descendants.

Two graphs with the same skeleton and the same set of V-structures (immoralities), are I-equivalent graphs [1]. The group of I-equivalent graphs can be expressed as a PDAG. However, the opposite does not hold, graphs of the same I-equivalent group (and same skeleton) may well have a different set of V-structures. Therefore, knowing the skeleton and the V-structures set, only a PDAG can be unequivocally built. In order to build a DAG from the PDAG, one of the multiple solutions from the PDAG must be chosen. The process of orienting edges depends on the node ordering; taking into account that a graph with *n* nodes has *n*! different node permutations, two main options are proposed to overcome this computational explosion:

- Ordering the nodes once again by mutual information, which gives two possibilities to orient the edges: orienting by the weakest first (TWF) or the strongest first (TSF).
- Creation of the DAGs using random node orders, and then calculation of the likelihood score for each DAG, choosing that DAG with the best likelihood score. In particular, we made use of a log-likelihood function with BIC regularization to penalize graph complexity.

For the assessment of BNs we have preferred a probabilistic approach based on goodness-of-fit, instead of graph recovery errors. The graph recovery errors were considered to assess all the CI-Maps since CI-Maps are formed only by undirected edges, where there exists an optimal skeleton that represents the conditional dependence. The errors are divided into two kinds: excess of edges (FP) and lack of edges (FN). However for BNs, the graph recovery errors in a DAG are divided into three kinds: FP, FN and the edge direction. And for the edge direction there is not a unique valid solution in terms of expressing the joint probabilities as factorised conditional probabilities. For instance:

- *P*(*A*, *B*, *C*) = *P*(*C*|*B*, *A*)*P*(*B*|*A*)*P*(*A*) ⇒ *A* → *B* → *C*
- *P*(*A*, *B*, *C*) = *P*(*A*|*B*, *C*)*P*(*B*|*C*)*P*(*C*) ⇒ *A* ← *B* ← *C*

Both expressions are equivalent in terms of measuring the goodness-of-fit (likelihood score), but only one would be correct in terms of DAG errors. The exception would be in the amount of V-structures created (*A* → *B* ← *C*), some of them are identified by the PDAG (PC-algorithm outcome when CI-Map is created), and others are an excess of V-structures created when undirected edges are oriented to form a DAG. Because the measurement of the amount of wrong V-structures does not strictly depend on the edge orienting order, and finding the true DAG is impossible from a PDAG, the likelihood approach was eventually chosen.

In any case, DAG recovery errors are reported in Figs 6, 7, 8 and 9. These errors are averaged from one random DAG associated to each CI-Map. The figures show that there is no correlation between DAG errors and sample size, there is a constant offset between (DAG—TOTAL) errors, where TOTAL is referred to the skeleton structure (FP + FN).

The definition of the BIC score used in the paper is extracted from [1]:

$$\begin{array}{c} {\text{BIC}}_{score}=\sum_{i}^{N}\text{ln}(P(X_{i}|\;parents\left(X_{i}\right)))-\frac{\text{ln}(N)}{2\cdot {Dim}_{G}} \end{array}$$ (8)

where *N* is the sample size, *Dim*_*G*_ is the model dimension, i.e. the number of independent parameters in the graph, *G*. The higher score the better goodness-of-fit, as its values are negative due to the logarithm of probabilities.

### 3.4 CI-Maps as feature selection

The analysis of the CI-map associations (edges) in relation to the target variable allows the identification of the features (nodes) that are associated with the target variable, considering the first or even the second order edges as the influence neighbourhood of node dependence.

Building the CI-map using the PC-algorithm with the set-up developed in this work, allows us to obtain a skeleton where we can identify the variables that are connected with our target variable, but the robustness of this CI-map is unknown, i.e., the representativeness of the variable associations is not known. To assess the robustness, a possible solution is to create a set of CI-maps from bootstrapped data, i.e. re-sampling with replacement keeping the same dataset size. These new bootstrapped CI-maps should have a similar skeleton with little variations in the edges.

An implicit measurement of edge robustness can be achieved by analysing the frequency of first/second order connections between the target variable and nodes identified as having the most frequent associations. From the collection of CI-maps generated by data bootstrapping, a hierarchical filtering is proposed in which maps are filtered sequentially following the order of most frequent connections in relation to the target variable, in such a way that, at the end, the filtered CI-map is the most representative map of the collection.

With this final CI-map, feature selection can be achieved by only considering the features connected to the target variable. Depending on the purpose of the analysis, this can include only first order or also second order associations. This analysis is always made in relation to a target variable; if the target changes, the most representative CI-map will likely be different.

Recalling that CI-maps require categorical variables, any continuous features have to be categorized, usually by quantiles. However, this affects the number of total categories and, in turn, the degrees of freedom of the independence tests, which are based on *G*^2^ statistic. There is a rule of thumb where the *G*^2^ statistic is not a suitable approximation of the *χ*^2^ distribution if the ratio between the sample size and the degrees of freedom falls below 5, hence, the requirement is:

$$\begin{array}{c} \frac{\text{sample}\;\text{size}}{DoF}\geq 5 \end{array}$$ (9)

Therefore, the degrees of freedom need to be controlled by adjusting the number of categories for continuous features. Depending on the sample size, this can be achieved simply by controlling the quantiles or merging categorical data into larger groups.

## 4 Data

### 4.1 Benchmark data for validation

Two well-known datasets were used for benchmarking, namely: Insurance and ALARM datasets, available in the Bayesian Network Repository (http://www.cs.huji.ac.il/~galel/Repository/). The Insurance dataset is a Bayesian network for evaluating car insurance risks [20]. It has 27 nodes, 52 edge degrees, 984 parameters, 5.19 average Markov blanket size, 3.85 average degree and 3 maximum in-degree, being the edge degrees the number of incident edges in a node, the in-degree the number of edges pointing to the same node (this form is also known as a V-structure), and the Markov blanket of a node is defined by the set of neighbouring nodes that isolates or shields the node from the rest of the network.

The ALARM dataset is a network designed to provide alarms during patient monitoring in anaesthesia [21]. The name stands for *A Logical Alarm Reduction Mechanism*. This data has 37 variables (nodes), 46 edge degrees, 509 parameters, 3.51 average Markov blanket size, 2.49 average degree and 4 maximum in-degree.

Knowing beforehand the probabilities that describe these Bayesian Networks, it is possible to generate sample data of different sizes. In this work, sample sizes from 0.5k up to 100k observations were used.

### 4.2 Brain tumour MRS data

The capabilities of our proposed methodology is tested against a real-world application, namely, a brain tumour MRS data, with the purpose of elucidating and understanding the associations between brain tumour types and a range of observed metabolites. Improving the knowledge and understanding of these associations is a relevant matter, since the anatomical information provided by magnetic resonance imaging (MRI) is not always accurate, especially in case of heterogeneous, glial tumours [22–24]. Although high resolution magnetic resonance techniques with tumour samples are described in the literature for validating *in vivo* MRS findings [25, 26], this requires invasive (surgical) obtention of additional samples from patients, since tissue used for histopathology analysis is not suitable. Other challenges associated with these approaches are the post-mortem changes and the different magnetic fields/resolution applied, giving rise to signals that are not feasible to observe *in vivo*. Altogether, this reinforces the interest of exploring the association between brain tumour types and metabolites *in vivo*, which reflects the type of data radiologists/technicians will face in their clinical practice, avoiding the need of invasive procedures which are not exempt of morbidity.

The dataset used was extracted from INTERPRET, an international multi centre database [27] resulting from the INTERPRET European research project [28]. It was formed by 217 patients with different types of brain tumours, and 22 healthy (control) individuals. Class labelling was obtained by histopathological analysis of a biopsy sample according to the World Health Organisation system for diagnosing brain tumours. The used data are short echo time (20-32 ms), single-voxel proton MRS (SV-^1*H*^-MRS) acquired at 1.5T and processed/postprocessed following the procedures described in [27].

The different tissue types were grouped into categories/classes, according to previous works [28, 29], as follows: meningiomas (class 1), aggressive tumours (class 2, grouping glioblastomas and brain metastases), and low grade glial tumours (class 3, including astrocytomas grade II, oligodendrogliomas, and oligoastrocytomas). We also added the normal parenchyma to this set of classes, as a fourth class. More details on them can be found below (the brackets indicate the number of patients/subjects in each class):

- Class 1, Meningiomas:
  - Meningiomas of WHO grade I (mm, 58 subjects)
- Class 2, Aggressive tumours:
  - Glioblastomas, giant cell glioblastomas and gliosarcomas (gl, 86 subjects)
  - Brain metastases (me, 38 subjects)
- Class 3, Low grade glial tumours:
  - Astrocytomas of WHO grade II (a2, 22 subjects)
  - Oligoastrocytomas of WHO grade II (oa, 6 subjects)
  - Oligodendrolgiomas of WHO grade II (od, 7 subjects)
- Class 4, Normal brain:
  - Normal brain tissue, white matter (no, 22 subjects)

These tumour groups are later processed as four independent binary variables, where each of them acts as target variable for the CI-map bootstrapping. Only one target is included in a CI-map to avoid the creation of spurious associations between tumour types which can mask associations with MRS-detected metabolites.

Although MRS can be performed with different nuclei such as ^1^H, ^13^C, ^19^F or ^31^P, the ^1^H-MRS is the one used in clinical settings. ^1^H-MRS provides insight into the biochemistry of examined tissues through a discrete signal in the frequency domain (obtained after Fourier transformation of time-domain signal) that reflects the relative abundance of several low molecular weight metabolites, lipids and macromolecules in the millimolar range. From each spectrum, a number of clinically relevant frequency intensity values, expressed in parts per million (ppm), were considered. The peak intensities (numerical variables) were categorized into three quantiles. This dataset has a small sample size and only three categories per variable ensure to fulfil the recommendation of Eq 9. The strategy was based on checking Eq 9 increasing the categories per sample, starting from two categories. Three categories per variable was the maximum possible according to Eq 8.

The following list compiles a selection of clinically relevant metabolites present in different types of brain tumours, and considered in this study:

- Mobile lipids (ML). The MR-visible lipids seen mostly at 1.28 ppm and 0.89 ppm are composed of triglycerides and cholesterol esters that accumulate in intracellular neutral lipid droplets. In human brain tumours, mobile lipids are observed predominantly in high grade tumours and in necrotic regions [30].
- Lactate (Lac) / Lipids. The main signal from lactate is a doublet arising from the methyl group, at 1.31 ppm, although another signal at 4.1 ppm can also be detected. The interpretation of the 1.31 main signal from lactate is complicated by mobile lipid overlapping [31] in short echo time spectra, being unravelled in long echo time spectra. Lactate is usually a product of anaerobic metabolism, being prominent in high grade tumours but accumulation in cystic or necrotic regions can produce increased levels independent of grade.
- Alanine (Ala). Alanine has its main signal as a doublet centred at 1.46ppm (Ala1), having also a signal at 3.77ppm (Ala2). Increased alanine has been observed *in vivo* in meningiomas [32].
- N-Acetyl aspartate (NAA). Singlet at 2.01 ppm is the most prominent resonance of NAA, although contribution of other N-acetylated compounds cannot be discarded [33]. It is considered as a neuronal marker, being its decrease associated with neuronal loss.
- Creatine (Cr) / Phosphocreatine (PCr). Creatine and phosphocreatine overlap in *in vivo* MRS and can be observed as a prominent singlet resonance from their methyl-protons, at 3.03 ppm [31], although other signals at 3.93 ppm may also be observed. This metabolite is related with cell energy cycles, being usually reduced in tumours in comparison with normal brain.
- Choline (Cho)-containing compounds. The choline signal is primarily observed as a prominent singlet at 3.21 ppm, which includes contributions from free choline, glycerophosphorylcholine, and phosphorylcholine which overlap *in vivo*, and it is often referred to as ‘total choline’ [31]. The choline signal reflects the turnover of cell membranes, being usually increased in brain tumours due to accelerated membrane synthesis in proliferating cancer cells [33].
- Taurine (Tau). Taurine can be seen as two triplets at 3.25 and 3.42 ppm. For *in vivo* studies at low field strengths, these resonances commonly overlap with the resonances from myo-inositol and choline.
- Glycine (Gly) / Myo-inositol (m-Ins). Glycine has two methylene-protons that co-resonate at 3.55 ppm. The glycine resonance overlaps with those of myo-inositol *in vivo*, making unambiguous observation of glycine not possible at shorter echo times [31], although a double echo time acquisition approach can estimate the differential contribution of both metabolites to a given spectrum [34]
- Glx group—including glutamate and glutamine (Glx). Glutamate (Glu) and Glutamine (Gln) are structurally similar, with two methylene and one methine groups and similar coupling patterns. Glu methine protons can be observed as a doublet-of-doublets centred at 3.74 ppm while Gln methine protons resonate at 3.75 ppm [31]. Other resonances for Glx can be observed at ca 2.14 and 2.35 ppm.

This summarizes the main signals (or group of overlapping signals) that are observed in *in vivo* MRS of brain tumoursand have special relevance for the classification proposed in this paper. Other minor signals relevant in specific scenarios such as PUFA (response to treatment) or 2-hydroxyglutarate (glioma subtyping) have also been described in literature, but it is beyond the experimental application described in this work to explore them, and these data were not acquired using dedicated MRS sequences to see these minor signals.

## 5 Methodology

### 5.1 Assessment of policies and parameters

First, the three options of the FDR policy are analysed to identify the procedure that produces the fewest skeleton errors in the benchmarked datasets. Then, the FDR is also tested with the FNR policy with the appropriate parameters in order to analyse the synergies between both policies.

As described in subsection 3.2, the FNR depends on the effect size, *w*. Therefore, the main objective is to determine, empirically, which is the range of *w* values that minimizes the total skeleton errors of the CI-map given the same sample size of the Insurance data. For this purpose, multiple CI-maps were retrieved by varying *w*. By plotting the False Positives, False Negatives and the total errors, the optimal *w* value that minimizes the total amount of skeleton errors could be identified.

The procedure to identify an appropriate value for *w* is then repeated for different datasets and sample sizes, where the variability of the appropriate *w* values against the sample size of the data is presented. The results are compared with those estimated in [10]. A final analysis of the FNR policy is carried out by plotting how the Insurance skeleton errors vary with respect to a wide range of sample sizes, where a fixed *w* value is considered as reasonably good since it cannot be estimated heuristically in the entire range of sample sizes. These experiments are repeated without FNR policy, whereby only the FDR policy is considered.

### 5.2 Node ordering in Bayesian network’s assessment

This subsection describes the methodology to assess a BN using a BIC score as a function of the node order. Two different node ordering approaches based on mutual information are proposed, and the BN generated with these predefined orders are compared with those generated by a random node ordering. The main goal of this subsection is to assess the score distributions of the three respective approaches to identify the protocol that, in general, gives the best BN.

The methodology consists in generating 100 samples of 25K observations of the Insurance dataset, having in mind that these samples can be generated because the probabilistic model is known for this problem. The aforementioned samples will produce 100 different CI-maps per data sample. Each CI-map provides a PDAG that can generate multiple compatible DAGs which represent the BN, where the generated BNs depend on the node order in which the edges are oriented.

The 100 BNs are scored with regard to the predefined orders by mutual information, the weakest first (TWF) and the strongest first (TSF). Then, for each data sample, multiple sets of BN are generated by random node order, and the ones with best and worst scores are selected, thus creating a distribution for worst/best score, respectively. The multiple sets are composed of 1, 25 and 100 different BNs with random node ordering. The three distributions are compared in the three-random-set scenarios.

The main purpose in this part of the study was to achieve a suitable baseline of a BN score with a predefined node order, and then run random node iterations until the BN score outperforms the best old score. The number of iterations depends on the computation time available. This approach provides a non-arbitrary baseline result for a realistic assessment of the performance of the respective policies.

### 5.3 Most representative CI-Map with bootstrapping

In this subsection, the methodology for highlighting the strongest features associated with the type of brain tumour is presented. The methodology starts with the pre-processing of the brain tumour MRS data, where the features are numerical variables obtained from the MRS within the spectral range in ppm associated with each metabolite. The metabolites are the main signals described in section 4.2. The signal features are discretized into categorical variables using three equidistant quantiles, i.e., percentiles at 33%.

The target variable is an external label, defined by the following four categories: low grade, aggressive, meningioma and normal, as explained previously (section 4.2). For the input of the CI-map, each category is considered as a binary variable isolated from the rest of the categories. Only one category is included per CI-map in order to obtain its feature associations without interference from other categories. Therefore, the process will be repeated four times, one per category.

The next step is to generate 400 bootstrapped data creating 400 CI-maps. This step is not computationally expensive for this size of data and, at the same time, it allows to obtain robust results. The results are recorded in an accumulative list of the first and second order associations connected to the tissue category. From this list, two histograms are constructed in order to visualize the most frequent associations for the first and second order connections of the target variable.

Inspecting the histograms provides qualitative insights about the structure of the CI-maps. For instance, if two nodes have complementary percentages between the first and second order edges, they probably are swapping positions in the edges. If in the first order histogram there are few nodes but with high percentages, this means the connections are very significant and most of the CI-maps are quite similar. On the other hand, if there is a number of nodes connected to the target variable, this means that there are many weak connections where edge variation is considerable, probably indicating that there are a number of weak connections and these nodes can appear as 1, 2 or 3 order connections, or even have no connection whatsoever (which indicates independence).

The next step in the procedure is to apply the hierarchical filtering, starting with the first order connections and selecting the most frequently connected node, then the second order and so on, until there is only one remaining CI-map or the rest are equal. In fact, after the third order connection we can discard the filter and just select one random node of the remaining maps. Nodes with a frequency less than 10% are ignored to avoid considering maps with low prevalence.

The selected CI-map is the most representative one in terms of the frequency of appearance. This procedure is a robust method whereby the target variable connections represent the strongest, most reproducible dependences. The resulting CI-map can then also be used to build a BN following the methodology described in subsection 5.2. The CI-maps identified using this approach are presented in the results of the paper from section 6.4 onwards.

### 5.4 Algorithm pipeline in pseudo-code

The pseudo-code of the followed pipeline is described in algorithm 1, where two main procedures are detailed: the first one, called *CImap2DAG*, builds a CI-map and transforms it into a DAG. The second procedure, called *MostRepresentativeCImap*, finds the most representative CI-map, and it is applied when one wants to analyse the main connections of a target-node. The input data is composed by categorical variables in columns (nodes) and observations in rows that follow certain correlations (edges). The first procedure returns the DAG with the best BIC score and the data column order after the specified maximum iterations. The second procedure returns the most representative CI-map with respect to the target-node, this CI-map can be used again as input for the first procedure to obtain its DAG, BIC score and column order.

**Algorithm 1** CI-map algorithm pipeline with recommended setup

1: data: collection of records (rows) described by categorical variables as nodes (columns)   ⊳ columns with no predefined order

2: **procedure** CImap2DAG(Input, FDR = basic, FNR = disabled, max-iter)

3:  **if** *type*(Input) is *data-type* **then**

4:   data ← **ColumnReordering**(Input, policy = TWF) ⊳ Columns sorted by decreasing unconditional mutual information

5:   CImap ← **PCalgorithm**(data, FDR = basic, FNR = disabled) ⊳ The weakest edges are pruned first

6:  **else if** *type*(Input) is *CImap-type* **then**

7:   CImap ← Input

8:  **else**

9:   *raise ERROR* ← *Input-type* must be *data-type* or *CImap-type*

10:  **end if**

11:  DAG_*TSF*_ ← **OrientingDAG**(CImap, policy = TSF) ⊳ The strongest edges are oriented first

12:  BIC_*TSF*_ ← **BICLogLikelihood**(DAG_*TSF*_)

13:  col-order_0_ ← **GetColumnOrder**(DAG_*TSF*_)

14:  DAG_0_ ← DAG_*TSF*_

15:  BIC_0_ ← BIC_*TSF*_    ⊳ This is the baseline score

16:  **for** iter ← 1: max-iter **do**

17:   DAG_*rnd*_ ← **OrientingDAG**(CImap, policy = random)

18:   BIC_*rnd*_← **BICLogLikelihood**(DAG_*rnd*_)

19:   **if** BIC_*rnd*_ > BIC_0_ **then**

20:    col-order_0_ ← **GetColumnOrder**(DAG_*rnd*_)

21:    DAG_0_, BIC_0_ ← DAG_*rnd*_, BIC_*rnd*_

22:   **end if**

23:  **end for**

24:  *return* (DAG_0_, BIC_0_, col-order_0_)

25: **end procedure**

26: **procedure** MostRepresentativeCImap(data, target-node, max-iter)

27:  CImap-list ← Empty list

28:  **for** iter ← 1: max-iter **do**

29:   temp-data ← **BootstrapData**(data)

30:   temp-CImap ← **PCalgorithm**(temp-data, FDR = basic, FNR = disabled)

31:   CImap-list ← Append(temp-CImap)

32:  **end for**

33:  hist-node ← **TargetNodeHistogram**(CImap-list, target-node)  ⊳ Get most frequent connections to target-node

34:  node-list ← **sorted-nodes**(hist-node, max-node-number)  ⊳ List of most prevalent nodes connected to target-node, until a max-node-number

35:  CImap-list_0_ ← CImap-list

36:  **for** *node*_*i*_ ← node-list **do**

37:   CImap-list_0_← **FilterCImapSubset**(CImap-list_0_, *node*_*i*_)  ⊳ Keep only those maps where *node*_*i*_ is linked to target-node

38:  **end for**

39:  CImap ← **SelectAnyFromList**(CImap-list_0_)

40:  *return* CImap

41: **end procedure**

## 6 Results

Subsections 6.1, 6.2 and 6.3 show influence of FDR, FNR and node ordering in a known network, Insurance. The assessment is carried out by counting the errors in the skeleton compared to the true structure. An error is considered as having: too many edges (FP), missing edges (FN), and the wrong edge direction in the case of a DAG. The BNs formed by DAGs are also evaluated using a log-likelihood score function with BIC as a regularization term.

### 6.1 False discovery rate

Table 1 shows the skeleton errors committed in a sample of 500 observations of the Insurance dataset, for each of the FDR control policies. The FNR setup used had an effect size of *w* = 0.25, power of *β* = 0.05, and the option of ordering nodes by the weakest first (TWF) was enabled. Results improved when the FNR was activated, showing a decrease in False Positives (FP) but no significant changes were observed with respect to the FDR.

**Table 1:** Insurance average skeleton errors.

SETUP	*FN*	*FP*	*TOTAL*
*FDR basic*	3.1 ± 1.2	25.3 ± 1.5	28.4 ± 2.5
*FDR interleaved*	3.0 ± 1.5	25.2 ± 1.5	28.2 ± 2.8
*FDR mini*	2.8 ± 0.9	25.0 ± 1.6	27.8 ± 2.4
*FDR basic + FNR*	3.3 ± 0.9	20.6 ± 1.6	23.9 ± 1.9
*FDR interleaved + FNR*	3.4 ± 1.3	20.4 ± 1.4	23.8 ± 2.4
*FDR mini + FNR*	3.4 ± 1.1	20.4 ± 1.4	23.8 ± 2.3

Averaged skeleton errors for 10 samples of the Insurance data with 500 observations each.

These results were compared with the R package PCALG [35], which is a well-known library for R-language that implements the PC-algorithm among other structure-learning algorithms. The purpose of this comparison was to check whether the results can be considered similar and consistent. Using the same 10 samples of the Insurance dataset with 500 observations, Table 2 shows the skeleton errors of three structure finding algorithms from the PCALG package: the Fast Causal Inference (FCI) algorithm [5], and two variants of PC-algorithm [14], namely special and relaxed. The special variant tests that no additional V-structures are needed if a DAG is built, whereas the relaxed variant does not apply this condition.

**Table 2:** Comparison with PCALG R package.

PCALG algorithms	Skeleton errors	
	500 obs.	5000 obs.
FCI	26.6 ± 1.1	24.7 ± 1.5
PC special	25.7 ± 1.0	22.6 ± 2.4
PC relaxed	25.7 ± 1.0	23.2 ± 2.4

PCALG R package Insurance CI-map comparison for 500 observations. Additionally, results with 5000 observations have been added to show how the errors decrease with the sample size.

The results are quite similar between them, where their sample standard deviation cover the mean errors of the different groups. The mean skeleton errors in decreasing order are FDR without FNR (28.1 ± 2.6), PCALG (26.0 ±1.0) and FDR with FNR (23.8±2.2).

### 6.2 False Negative Reduction

**Fig 1:**
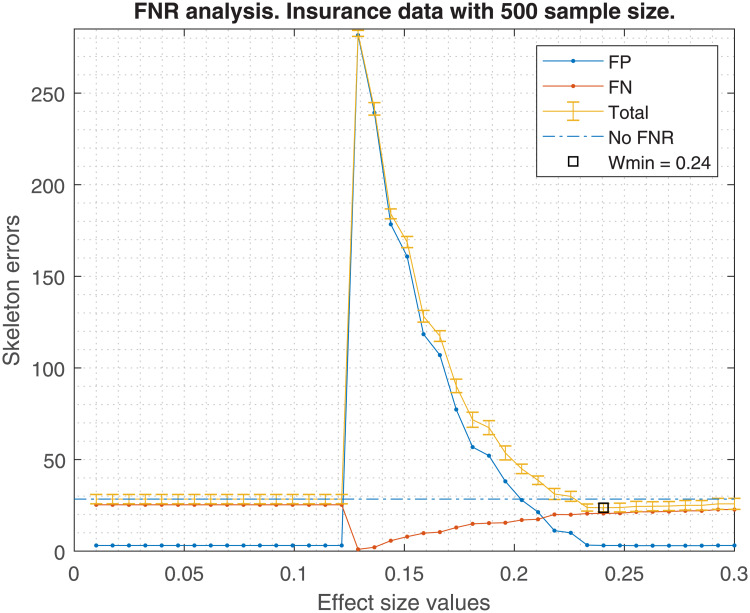
Insurance structure errors. The figure shows the averaged structure errors for Insurance BN with 500 observations, applying the FNR policy.

**Fig 2:**
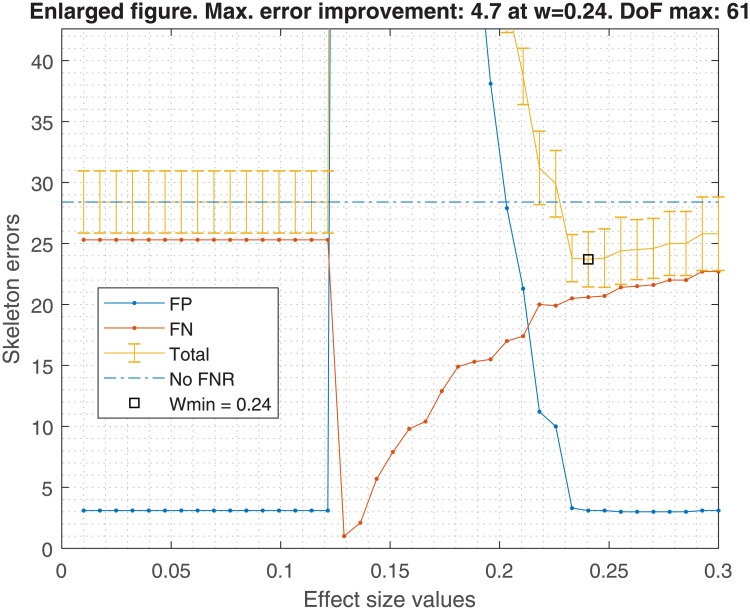
Zoomed Insurance structure errors. The figure is a zoom in the critical point. With the optimal effect size, *w*_*min*_ = 0.24, a reduction of 4.7 skeleton mean error is achieved. However, the *w* range where the skeleton errors are improved with respect to not applying the FNR policy is quite narrow and close to those *w* values that produce a high FP proliferation. There are 61 degrees of freedom for the *w*_*min*_.

**Fig 3:**
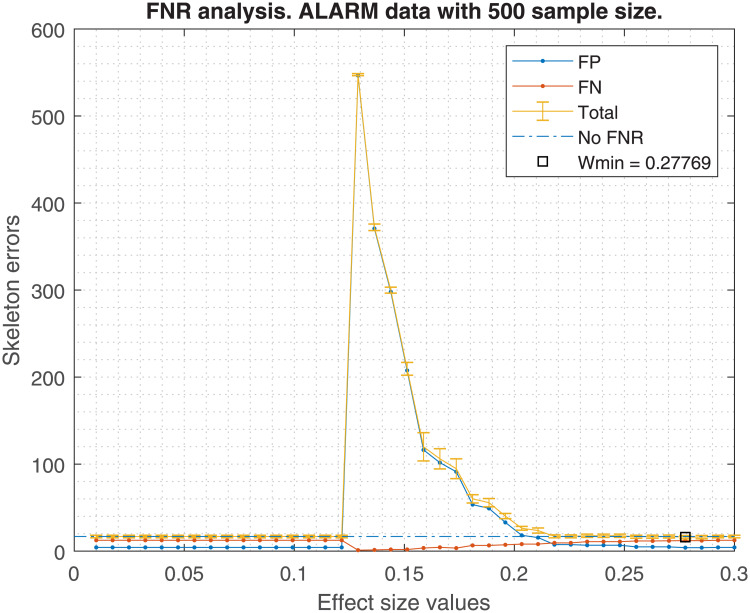
Alarm structure errors. The figure shows the averaged structure errors for ALARM BN with 500 observations, applying the FNR policy.

**Fig 4:**
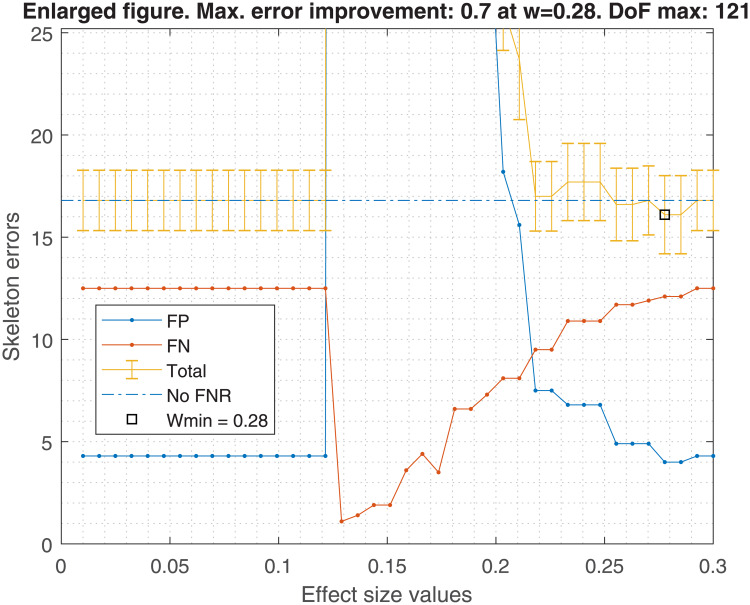
Zoomed alarm structure errors. The figure is a zoom in the critical point. With the optimal effect size, *w*_*min*_ = 0.28, a reduction of 0.7 skeleton mean error is achieved. However, the *w* range where the skeleton errors are improved with respect to not applying the FNR policy is quite narrow and close to those *w* values that produce a high FP proliferation. There are 121 degrees of freedom for the *w*_*min*_.

Figs 1, 2, 3 and 4 show the averaged skeleton errors of 10 different samples of Insurance and ALARM dataset respectively, both sets have 500 observations and the FNR policy is applied. This helps illustrating the pattern of the structure errors with reference to the effect size *w*. In particular, we can see that the observed behaviour was similar for different sample sizes but displaced to the left (i.e. same peaks but with lower values of effect size).

A number of insights can be gained from the inspection of these figures:

- FN errors decrease as the effect size decreases until some point where all tests are avoided and the graph becomes fully connected. Beyond this point FNR is disabled.
- Similarly, the FN errors start to decrease when the FP errors start to increase until reaching the fully connected graph where the FP errors get a maximum value.
- There is an effect size window, *δw*, where the trade-off between FP vs FN is acceptable, and the total error decreases. This *δw* depends on the dataset and the sample size.
- For this example of 500 observations, one may observe that the optimal effect size is *w*_*min*_ = 0.24, improving the average total error by 4.7, considering FP and FN. This improvement diminishes as the sample size increases, being 4.7, 3.6, 3.3, and 2.3 skeleton errors for 0.5K, 1K, 5K and 10K samples, respectively. In the best case, an improvement of approximately 5 skeleton errors does not guarantee the high risk of selecting a bad effect size for new data.

**Fig 5:**
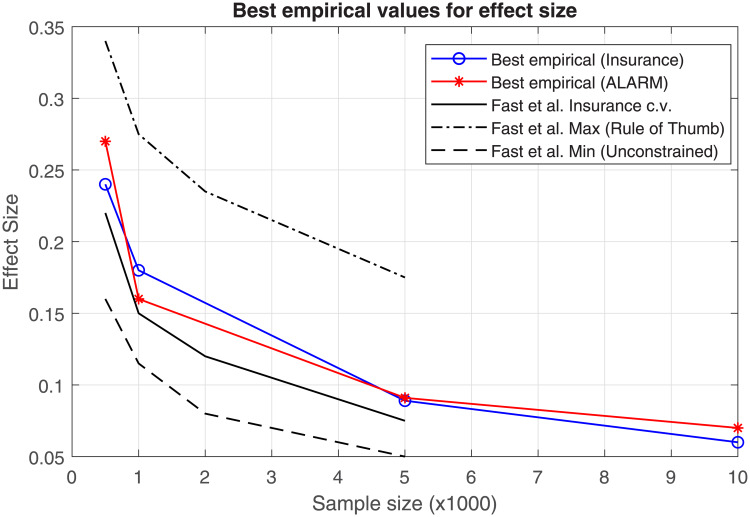
Empirical effect size values. This plot shows best empirical values for effect size parameter, *w*, with respect to the sample size. Our empirical values are compared with the ones of [10].

Fig 5 shows the optimal effect sizes, *w*, found through empirical tests with Insurance dataset network (blue line) and ALARM network (red line). The procedure used is similar to the one used in Figs 2 and 4, this time scanning several effect size values and selecting the one that minimizes the total skeleton errors.

Additionally, in the same plot one may observe (black lines) the effect size suggested by [10]; in that work, the optimal effect size is obtained from a random sample of Insurance network using a cross-validation at each sample size. Also a bandwidth of Δ*w* ≈ 0.15 is depicted, where the lower bound is formed by an unconstrained skeleton, and the upper bound is formed by a rule of thumb; the rule of thumb states that the independence test (G2-test) is reliable if there are five or more instances per degree of freedom of the test. If the test is not reliable, PC-algorithm defaults decision is to include the edge in the skeleton.

Using the suggested effect size for a 500 sample size in the Fig 5, we should obtain a *w* = 0.22. If that value is taken, the errors shown in Fig 2 would be higher than if FNR were disabled. An analogous situation occurs with Fig 4.

On the other hand, the bandwidth Δ*w* is too broad to bound a good *w* range within the empirical effect size window shown in Figs 2 or 4, in which the FNR policy helps to decrease the total skeleton errors. Hence, FNR should not be recommended for new datasets or if the effect size cannot be selected optimally.

**Fig 6:**
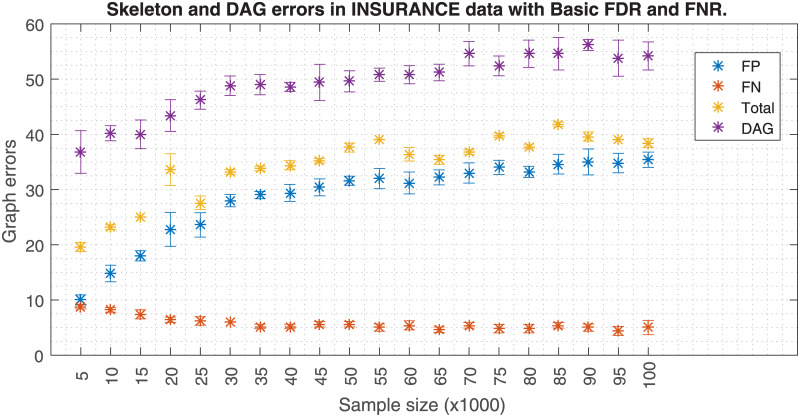
Insurance errors due to sample size with FNR. The figure shows the skeleton and DAG errors with respect to the sample size applying FDR and FNR policies for the Insurance dataset.

**Fig 7:**
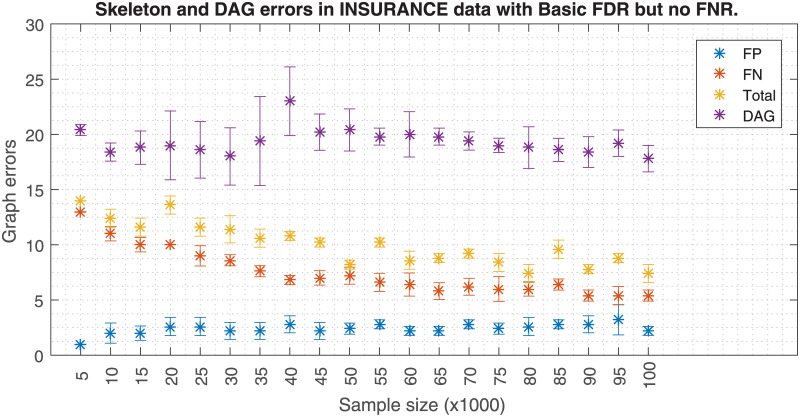
Insurance errors due to sample size without FNR. The figure is similar but without FNR policy. FNR policy needs *w* to be adjusted to the sample size; keeping *w* as the sample size increases (top figure) produces a FP proliferation. The skeleton errors of the bottom figure decrease asymptotically to ≈7.5. Also the DAG errors based on edge orientation are depicted in both figures.

**Fig 8:**
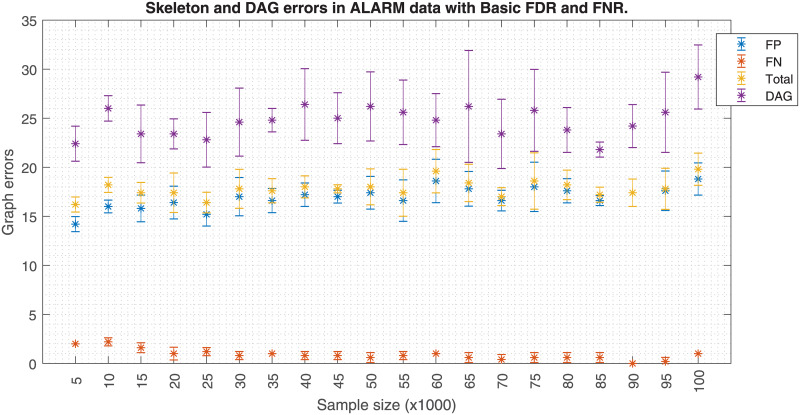
Alarm errors due to sample size with FNR. The figure shows the skeleton and DAG errors with respect to the sample size applying FDR and FNR policies for the ALARM dataset.

**Fig 9:**
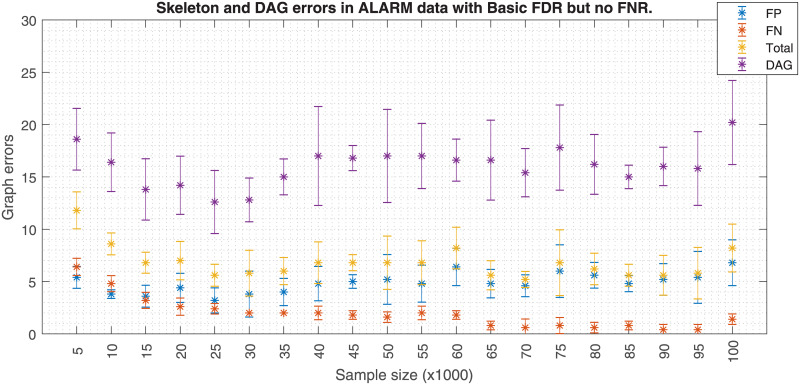
Alarm errors due to sample size without FNR. The figure is similar but without FNR policy. FNR policy needs *w* to be adjusted to the sample size; keeping *w* as the sample size increases (top figure) produces a FP proliferation. The skeleton errors of the bottom figure fluctuate around to ≈7. Also the DAG errors based on edge orientation are depicted in both figures.

Similarly, Figs 6 and 7 shows how a fixed effect size *w* affects the skeleton errors when the data size increases. When FNR is activated, an increasing FP error is observed when the data size increases. If FNR is deactivated a significant FP reduction is observed. Hence, these plots suggest that the effect size has to be carefully adjusted as a function of the sample size. Theoretically, the PC-algorithm would converge to the true structure if the whole population data were available. However, in a realistic case scenario like in Fig 7, the skeleton errors for sample sizes bigger than 100k asymptotically tend to a non zero value, ≈7.5. In this analysis, the errors for the DAG have a different source, which is related to the lack of a unique DAG solution once the skeleton is fixed. Since the application of the orienting rules of [11, 19] does not unequivocally solve the DAG, other studies make use of the PDAGs. An analogous situation occurs with Figs 8 and 9 but the FP errors are more stable than in the case of the Insurance dataset when FNR is disabled.

### 6.3 Results about the effect of node ordering

**Fig 10:**
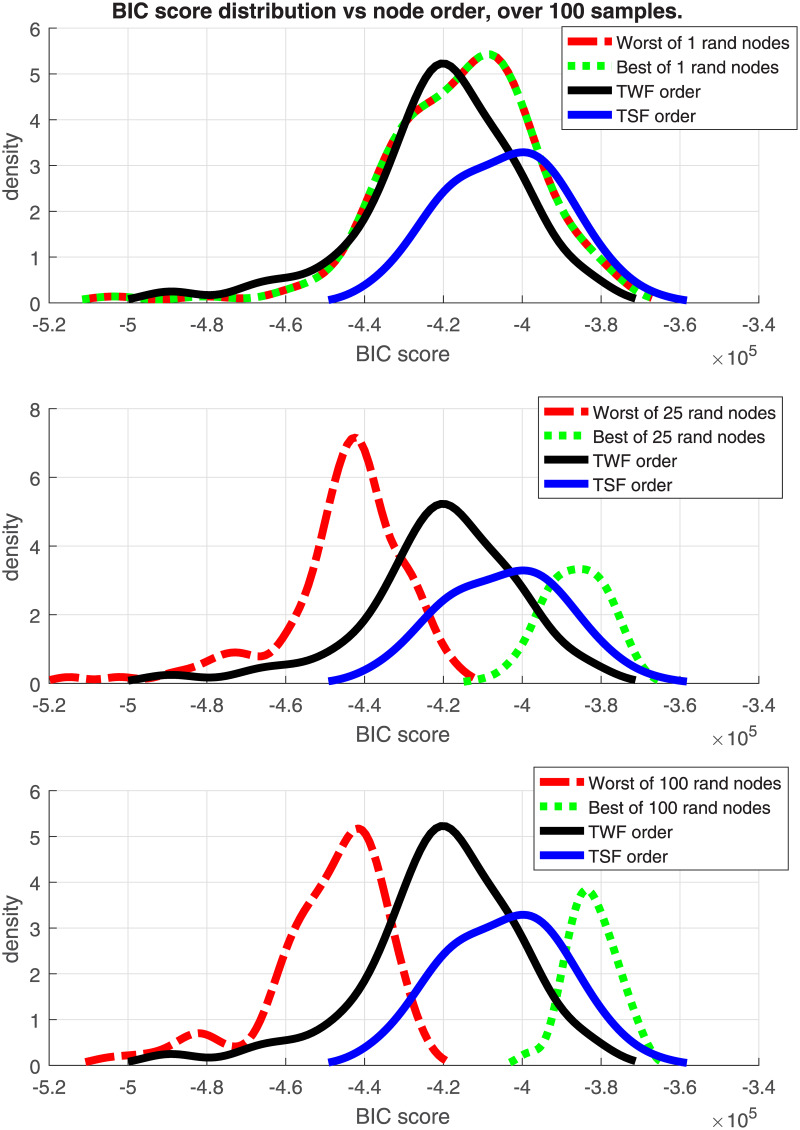
Insurance BIC score distributions. BIC score of node order distributions for the Insurance network.

**Fig 11:**
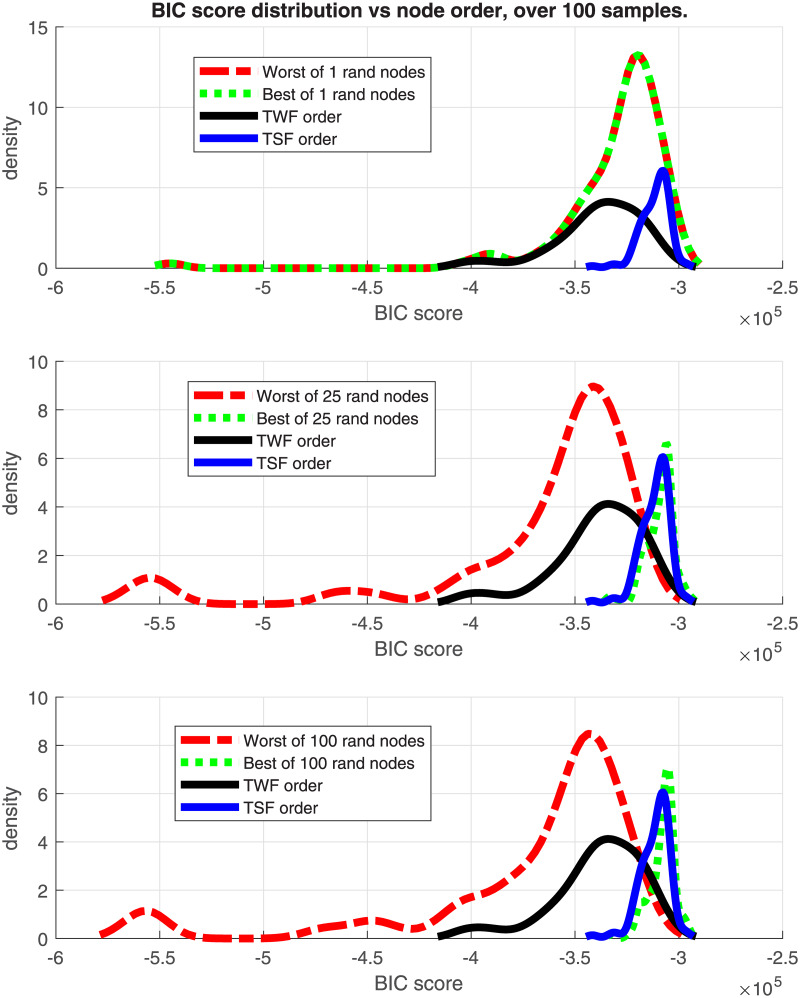
Alarm BIC score distributions. BIC score of node order distributions for the ALARM network.

Figs 10 and 11 show the density functions of the BIC score, using a non-parametric kernel-smoothing distribution for 100 samples of the Insurance and ALARM dataset with 25K observations each, and 27 and 37 nodes, respectively. In each plot, there are four distributions with TWF and TSF node order, and the best and worst solutions obtained by random ordering. There are three scenarios: only one random order (being only one, worst = best), 25 random samples, and 100 random samples.

The graphs show that the TSF node order policy is the best option when there is a limitation in the number of repetitions for building a new DAG with random node order. Such a limitation may be, for instance, due to a computational time constraint for a large data set, or when the graph has too many nodes and a large number of repetitions would be needed to extract a representative sample of the node order permutations. In contrast, the score distribution of the TWF node order policy is centred in a region of lower scores and presents a long left tail. This tail can produce a poor DAG score depending on the sampled data. The tail of TSF distribution is shorter and it defines a lower bound for the DAG score.

When there is no runtime constraints for creating a new DAG sampling node order permutations, the best option to obtain a faithful DAG (highest score) is the random sampling option. The basic idea is to repeat the random samplings as much as reasonably possible, and choose the solution with the best score. Even in this case, the TSF solution can be useful as a baseline score to compare with the initial random solutions. In the example with 25K observations, we can consider that the sample represents the whole population for the insurance dataset quite well. Decreasing the number of observations would lead to noisier distributions.

### 6.4 Brain tumour MRS results

This subsection shows the results of the CI-maps applied to the four target variables based on tissue type categories with reference to the clinically relevant brain tumour metabolites.

**Fig 12:**
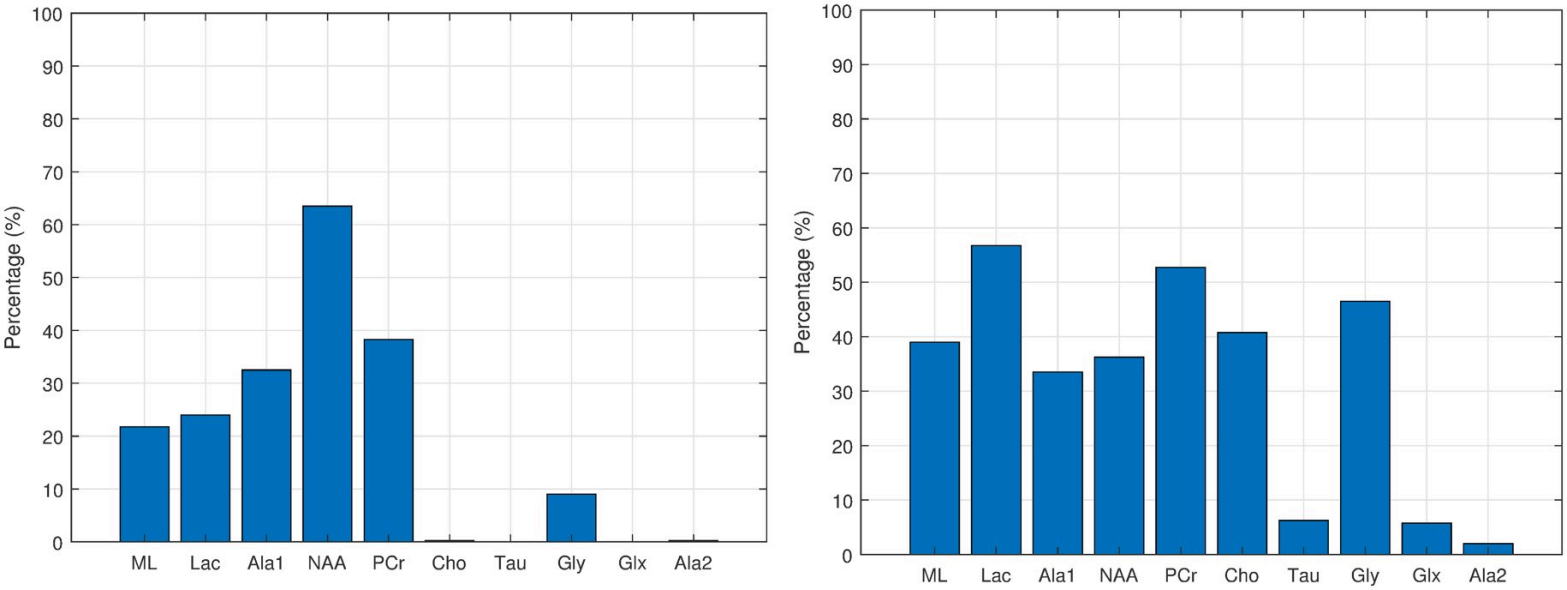
Associations of normal tissue. Histograms of the first (left fig.) and second (right fig.) order associations. Note the *Gly* bar also includes the *m-Ins* metabolite.

**Fig 13:**
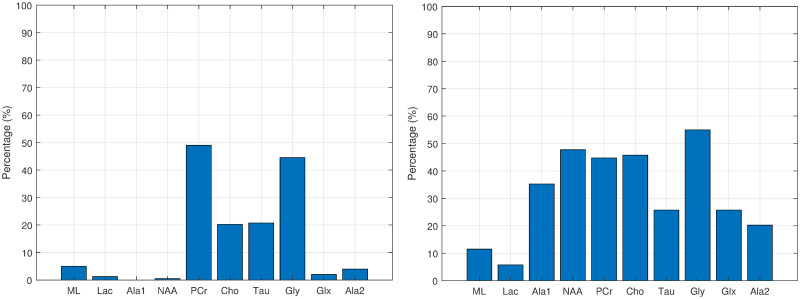
Associations of low-grade glial tumours. Histograms of the first (left fig.) and second (right fig.) order associations. Note the *Gly* bar also includes the *m-Ins* metabolite.

**Fig 14:**
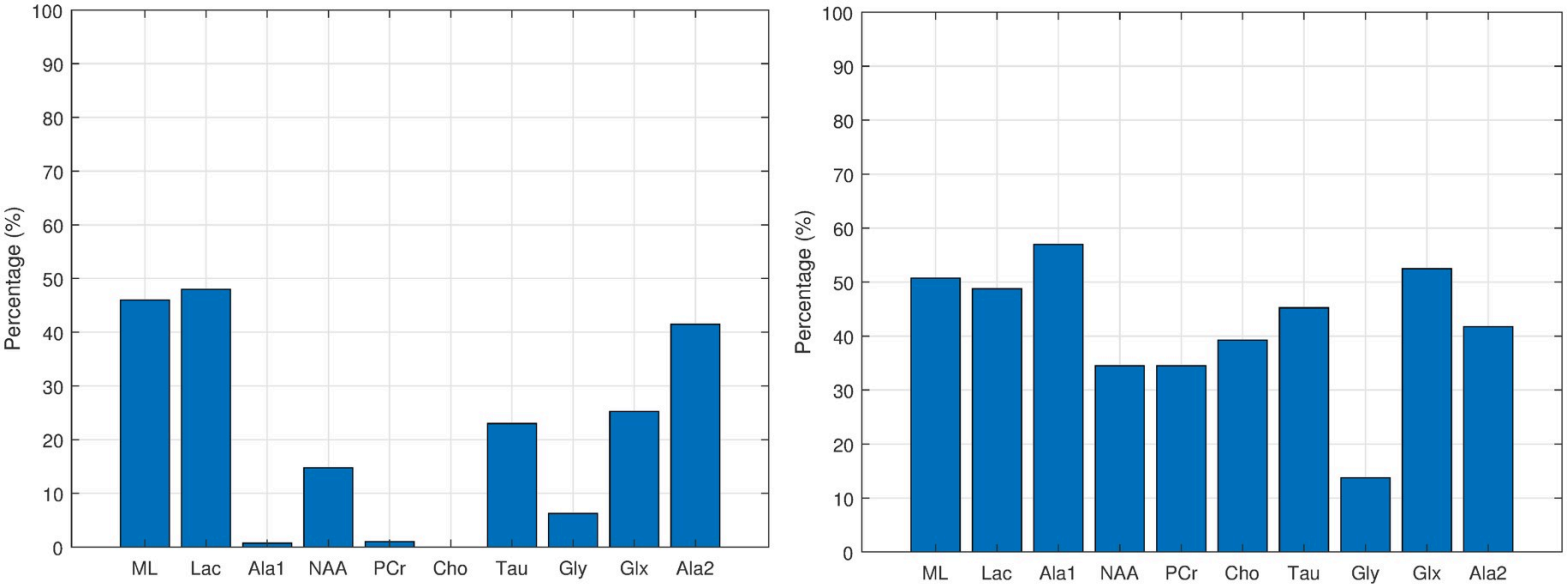
Associations of aggressive tumours. Histograms of the first (left fig.) and second (right fig.) order associations. Note the *Gly* bar also includes the *m-Ins* metabolite.

**Fig 15:**
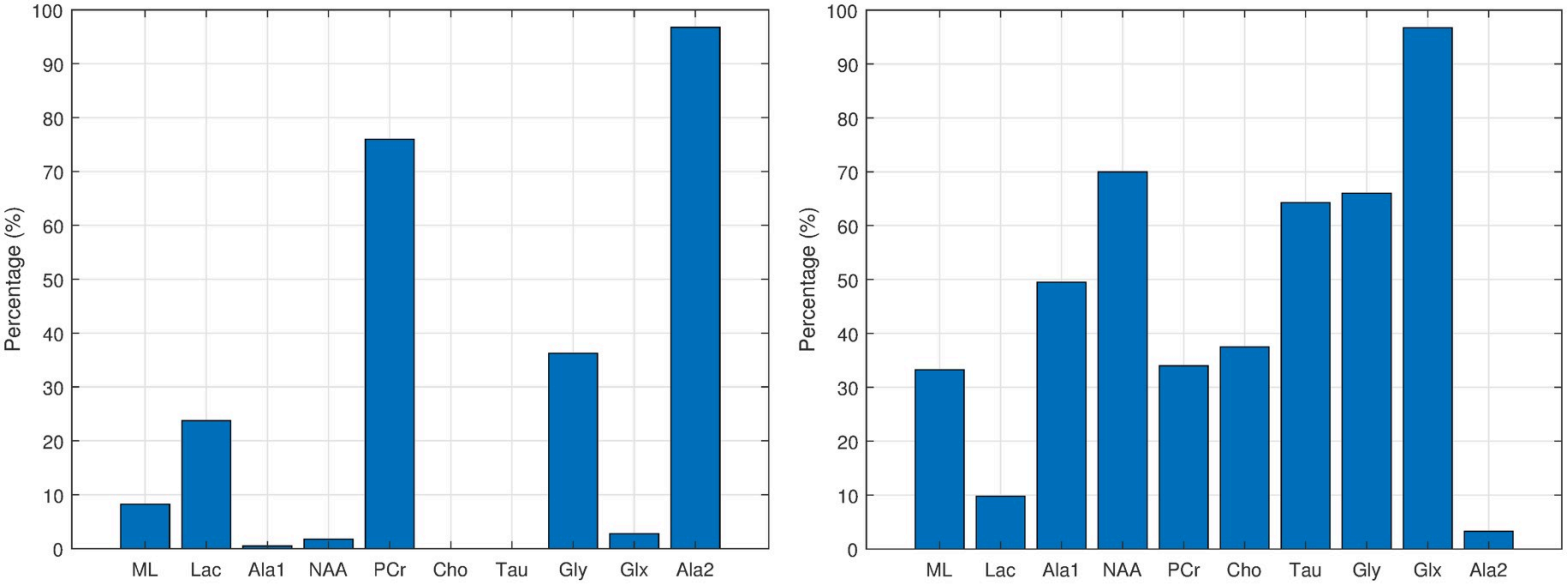
Associations of meningiomas. Histograms of the first (left fig.) and second (right fig.) order associations. Note the *Gly* bar also includes the *m-Ins* metabolite.

Figs 12, 13, 14 and 15 show the histograms of the first and second order associations related to the four tissue categories (target variables): Normal (healthy) tissue, low-grade glial tumours, aggressive tumours, and meningiomas. These were obtained from the bootstrapped CI-maps. In all CI-maps, the edges included the mutual information, the significance test was set to a p-value of 0.05, and the bootstrapping had 400 iterations.

**Fig 16:**
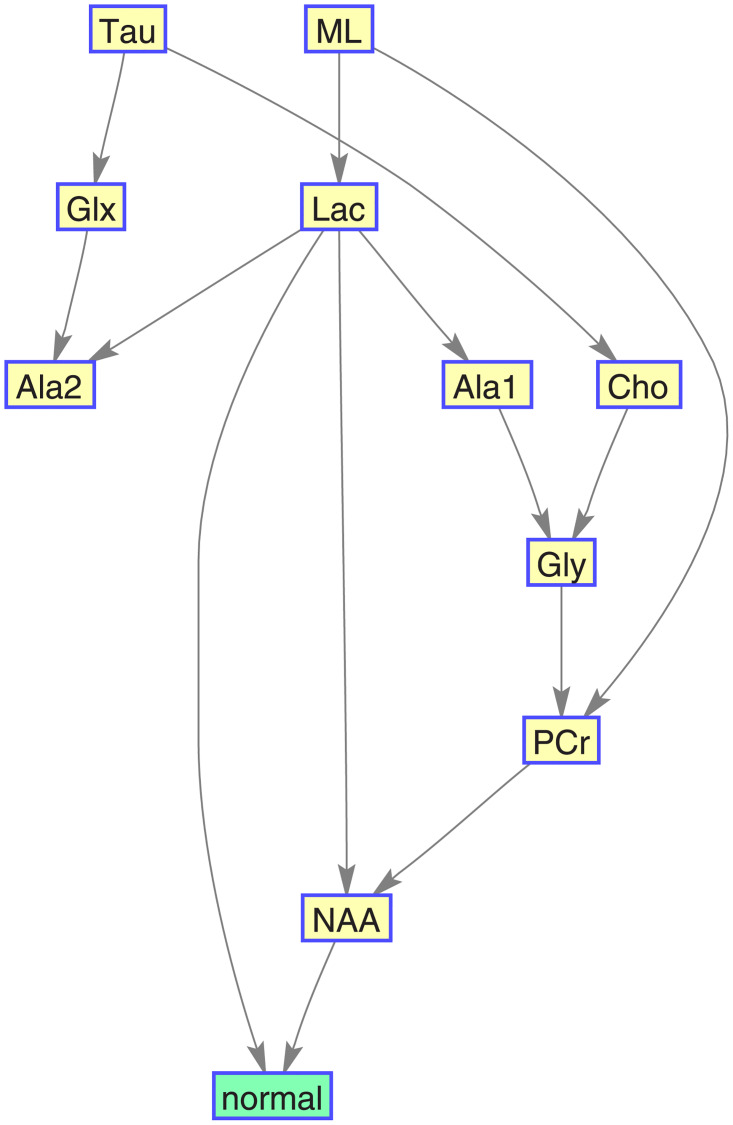
Normal tissue BN. It is based on its most representative bootstrapped CI-map.

**Fig 17:**
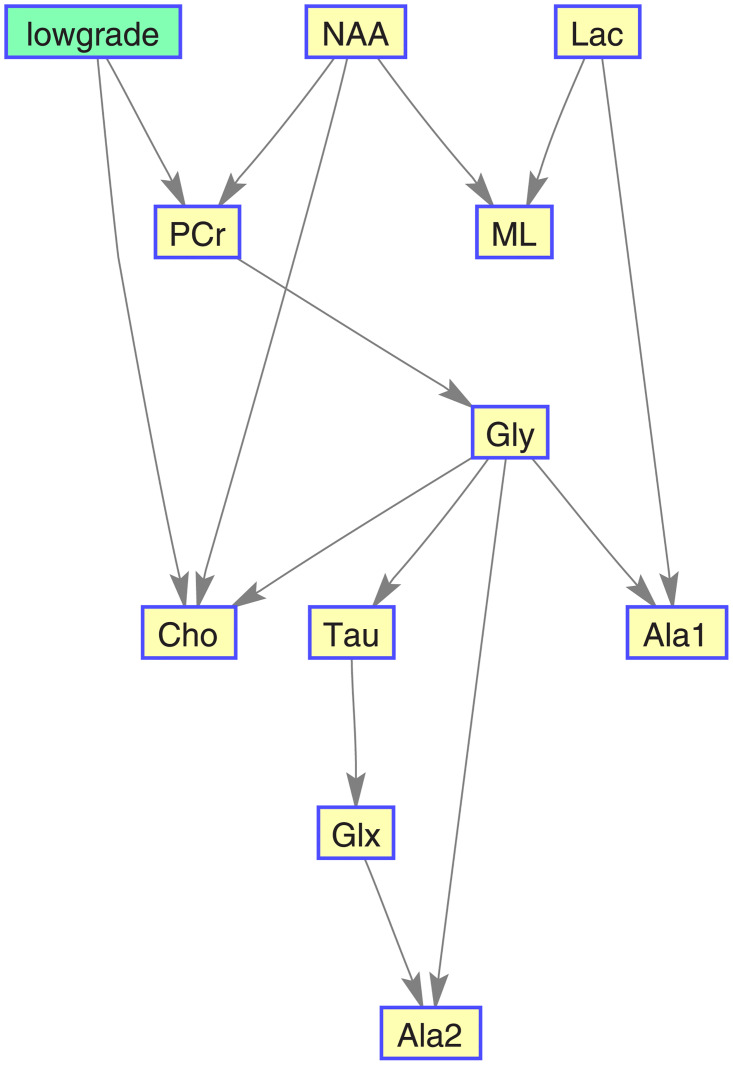
Low-grade glial tumour BN. It is based on its most representative bootstrapped CI-map.

**Fig 18:**
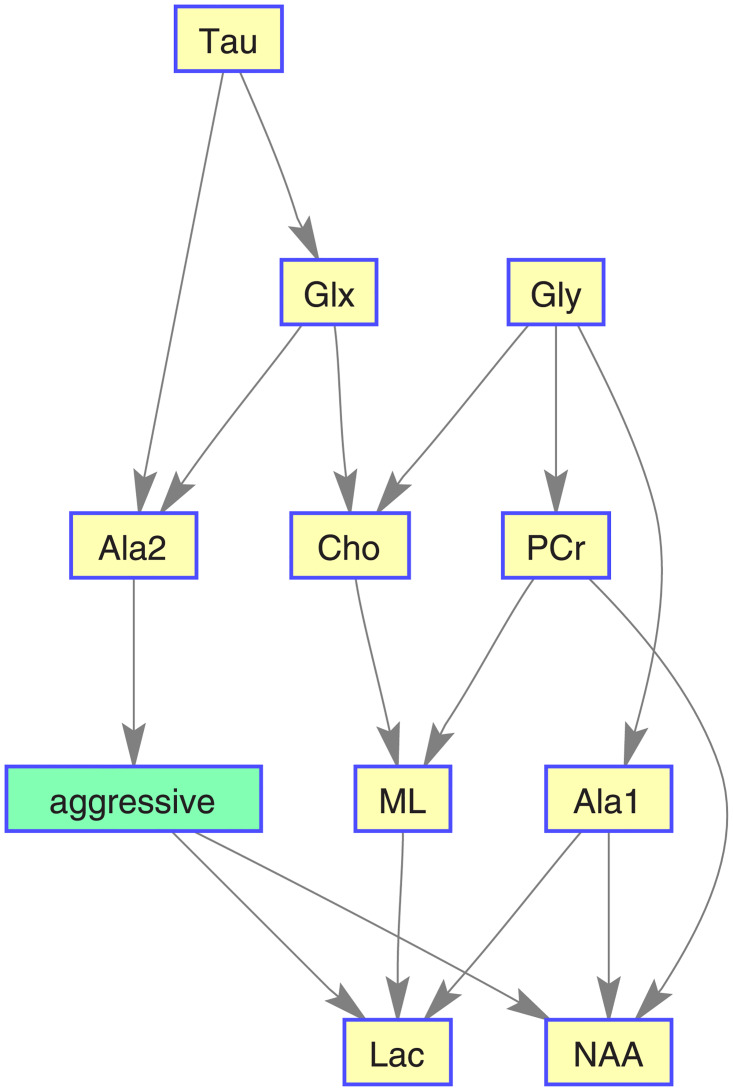
Aggressive brain tumour BN. It is based on its most representative bootstrapped CI-map.

**Fig 19:**
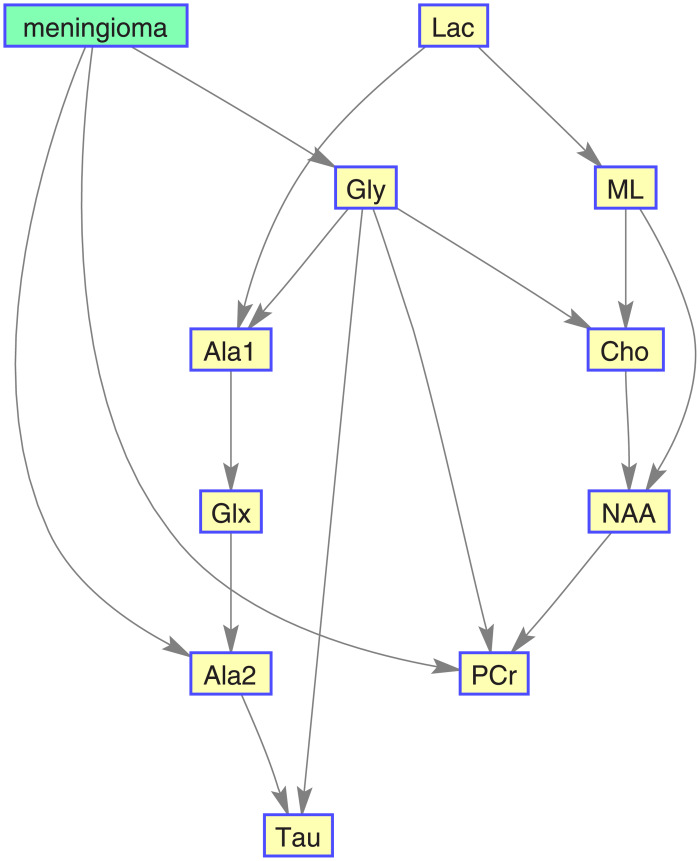
Meningioma brain tumour BN. It is based on its most representative bootstrapped CI-map.

Figs 16, 17, 18, and 19 show, for each target category, the Bayesian network derived from the bootstrapped CI-map applying the node order assessment methodology, starting with TSF node order and improving the BIC score with random iterations, in order to obtain the best BIC score. As mentioned before, the bootstrapped CI-map is a hierarchical filtered CI-map, which is the most representative CI-map of the bootstrapped data with respect to the most frequent node associations in relation to the target category.

**Fig 20:**
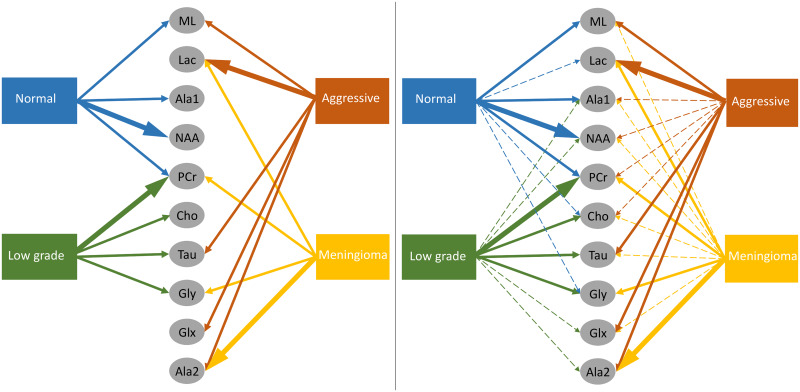
Brain tumour summary vs metabolites associations. The widest arrows represent the most frequent first order connections, the solid arrows are other first order connections and the dashed arrows are second order connections. On the left hand side only the first order connections are included, whereas on the right hand side also includes the second order connections.

The most important associations extracted from the prevalence histograms are summarized in Fig 20. The solid arrows connect the first order connections, the widest arrows indicate the predominant association, and the dashed arrows indicate second order associations. Note that a two-node association implies a correlation between these nodes (variables), but it can be positive or negative.

## 7 Discussion

### 7.1 CI-Map methodology

Some remarkable insights about the methodology and policies developed to build CI-maps by the PC-algorithm are:

- In terms of FDR policy, experiments performed in section 6.1 showed no significant differences in the structure errors between any of the three FDR policies proposed in [4]. Therefore, any FDR policy could be chosen, although the basic FDR was recommended because it has a simple implementation. This is without taking into account the FNR policy, suggesting that it would be safer to avoid its activation, discussed in the next point.
- The FNR policy was able to reduce some FP in the CI-maps shown in Table 1, but it required a very fine adjustment of its parameters (such as effect size, *w*). For unknown data, which is the most frequent situation, the suitable parameters are unknown, and an improper adjustment can produce an increase of FP as seen in Figs 2 and 4. The FNR parameters also depend on the sample size, which makes a proper adjustment be even more complicated. For that reason, it is safer to disregard the FNR policy.
- When a DAG is created from a CI-map, the node order for edge orienting influences the BN outcome based on the BIC score. Figs 10 and 11 show the BIC score distributions, where pre-ordering the nodes by strength of mutual information warrants a good baseline BIC score. However, it can be outperformed by BN with random node ordering, where it is necessary to perform as many iterations as practically possible, being 100 iterations a reasonable number to obtain a significantly better score distribution.
  Intuitively, the reason why TSF has a better performance than TWF is because TSF starts orienting the strongest edges first. This effect is just the opposite in the CI-map pruning process (edges cut), where the performance improves if the fully connected graph is being pruned starting from the weakest edges.
- If there is a target variable, bootstrapping methods can be applied to obtain a robust identification of the most frequent edges connected with the target variable. From the generated CI-maps, a hierarchical filter is applied to select the most reproducible CI-map. From the CI-map, a BN can be constructed.

### 7.2 Brain tumour and normal tissue CI-maps

In general, the metabolites associations with the tumour categories agree with the expected dependences described in the literature. For instance, normal tissue had strong first order association with NAA (neuronal marker), whereas aggressive tumours had strong correlations with ML and Lac, and meningiomas with Cho and Ala, as expected in representative datasets. Analysing each category in more detail:

- Normal tissue: Sorting in decreasing order, the associations with highest prevalence are NAA, PCr and Ala1, with 58%, 42% and 35%, respectively. The N-Acetyl aspartate (NAA) association is the expected result, being an abundant brain metabolite, predominant in normal brain tissue. Interestingly, the CI-map shows that there are other metabolites completely disconnected from normal tissue, like Tau, Ala2 and Glx.
  This apparent discrepancy between Ala1 and Ala2 signals could be due to the overlapping of other contributing metabolites at this spectral range such as Glycine or Glutathione [31] which altogether are not the main signals that distinguish normal from other brain tumour tissue categories.
  This apparent discrepancy between Ala1 and Ala2 signals could be due to the overlapping of other contributing metabolites at this spectral range such as Glycine or Glutathione (Govindaraju et al 2000) which altogether are not the main signals that distinguish normal from other brain tumour tissue categories
- Low-grade glial tumours: Sorting in decreasing order, the associations with highest prevalence are PCr, Gly/M-ino, Cho and Tau, with 52%, 39%, 22% and 20%. Here, the associations differ from normal tissue, with appearance of Gly/M-ino and Cho, and disappearance of NAA association. According to [34], the Gly/M-ino resonance is relevant in grading astrocytic tumours, and it agrees with this signal being associated with low-grade glial category.
- Aggressive: Sorting in decreasing order, the associations with highest prevalence are ML, Lac and Ala2, with 52%, 45% and 43%. According to the literature [36], the lactates and lipids (Lac) are associated with necrotic cells. In the selected CI-map, Lac is a second order connection with aggressive tumours through ML and Ala1.
- Meningioma: Sorting in decreasing order, the associations with highest prevalence are Ala2 and PCr with 98% and 80%. Alanine is a well-known signal indicator of meningiomas [32], which are located around meninges, and are usually benign and circumscribed, and PCr is usually described to be low or absent in these tumours, which agrees with the associations found. However, one of the most common increased signals in meningiomas, Cho, did not appear in the found associations.

Results obtained mostly show consistency with existent literature, reinforcing the potential of our approach to assess the correlation between brain tumour types and different metabolites prevailing in each environment [28, 34, 36–43]. This information can be of helpful to refine future studies using spectroscopic information, which is especially relevant when the anatomical (MRI) information is confusing (e.g. heterogeneous tumour appearance, early stages without contrast enhancement etc.). Still, in the future more difficult dichotomic classifications such as early treatment outcome in brain tumour treatment could be investigated.

## 8 Conclusions

The paper a) points out the instabilities that result from the application of the PC algorithm; b) proposes policies to make this work more consistently; c) applies the policies to a complex data set.

This paper has analysed the best setup for structure finding stabilization based on the PC-algorithm. Our results suggest that any of the FDR policies can be used to control the FP errors with similar outcomes; in this sense, the basic FDR has been selected due to its simple implementation. The FNR policy is focused on decreasing the FN errors, but it is recommended not to activate it when the optimal values of the effect size are unknown, which is the usual case for new data. The effect size parameter *w* in FNR policy may produce an increase of FP errors if the parameter is not properly adjusted to the data. Finally, when building a DAG from the skeleton, the node in which the edges are oriented affects the final DAG. Ordering the nodes by mutual information is based on orientating by the strongest first (TSF) order, which provided the best BIC score compared with a random ordering distribution. However, if the DAG is generated multiple times by sampling random node orders, the best solution of multiple random node orders outperforms TSF; even in this case, the best methodology is to make use of TSF as a baseline score followed by random orders to improve the score. The higher the number of samples, the better BIC score is obtained, at the expense of an increase in computational cost.

We have extended a previous work that claimed that ordering the nodes by mutual information (using the weakest first TWF) tend to reduce the skeleton errors of the CI-Map [4]; in particular, we have empirically shown with two benchmark datasets, that in the process of orienting the edges of a CI-Map to obtain a BN, ordering the nodes by mutual information (using the TSF) tends to obtain better BIC score distributions than random orders or TWF orders.

When investigating connections to a target variable, the CI-maps can be used for feature selection. With the use of bootstrapping techniques, robust results can be obtained that identify the most relevant nodes (variables) that connect to a target variable. Apart from the variable associations, this method can be used to select the most representative CI-map from the bootstrapped CI-maps, subsequently to be transformed into a DAG.

The CI-map methodology has been applied to MRS data of brain tumour and normal brain parenchyma, producing consistent results about the metabolites associated with different tissue types, mostly in agreement with the literature in this regard. With the bootstrapped CI-maps, histograms of the first and second order edges have been built, reflecting the prevalence of each association. The most important metabolite associations have been summarized in Fig 20.

## 9 Supplementary materials

### 9.1 Structure of the benchmark data used for validation

**Fig 21:**
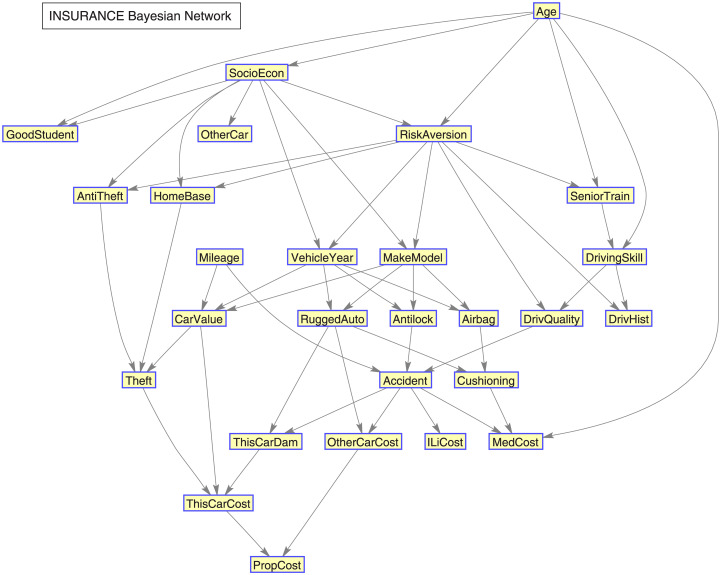
Insurance Bayesian network.

**Fig 22:**
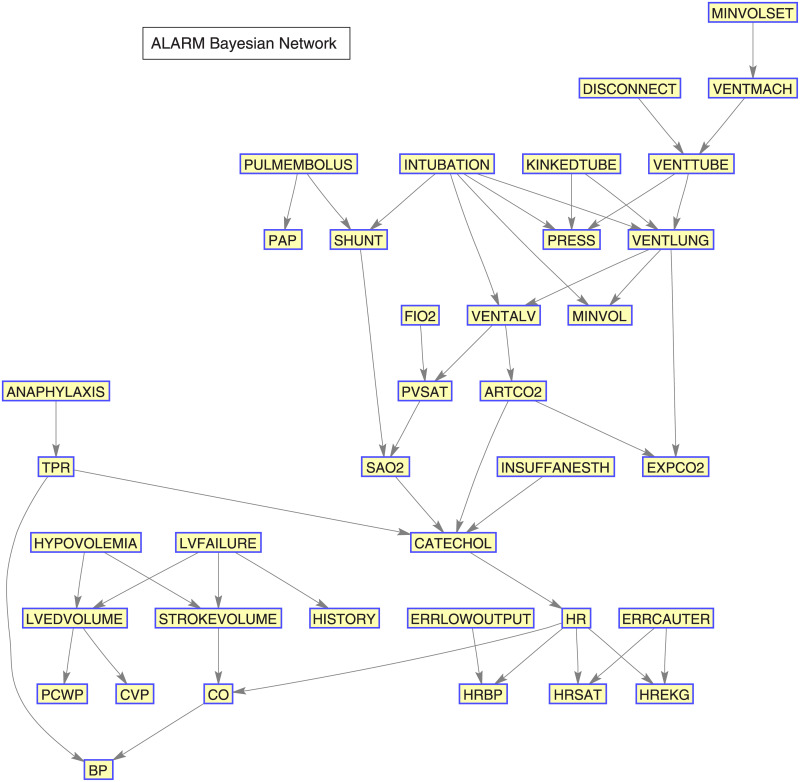
ALARM Bayesian network.

The structure of the Insurance dataset is presented in Fig 21, and the structure of the ALARM dataset in Fig 22.

### 9.2 Example of an original CI-map

**Fig 23:**
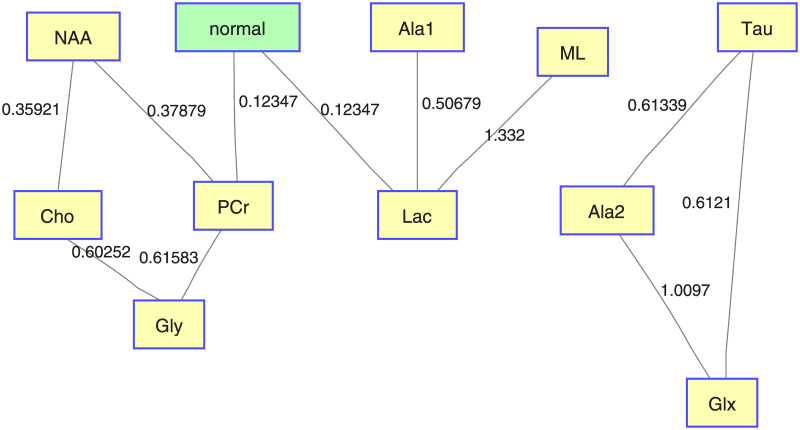
Original CI-map for normal tissue category. CI-map without bootstrapping. The edges indicate the mutual information between the nodes.

Here we include an example of a map (see Fig 23) obtained with the original CI-maps without bootstrapping, where edges represent the mutual information between the nodes. This example uses as target variable the normal brain (healthy tissue).
